# Association Between Low Vitamin D Levels, Sleep During Pregnancy and Post-Delivery, Depressive and Anxiety Symptoms: Longitudinal Analysis of the NiPPeR Trial

**DOI:** 10.1192/bjo.2025.10080

**Published:** 2025-06-20

**Authors:** Ciara McKay, Sheila Barton, David Baldwin, Keith Godfrey, Holly Austin

**Affiliations:** University of Southampton, Southampton, United Kingdom

## Abstract

**Aims:** Sleep is altered during pregnancy, particularly during the third trimester. Low Vitamin D levels have been linked to shorter sleep duration, depression and anxiety. In the NiPPeR double-blind randomised controlled trial of nutritional supplementation, women received either a formulation with additional ingredients including Vitamin D (‘intervention group’) or standard prenatal vitamins (‘control group’). The association between Vitamin D deficiency, sleep, depression and anxiety was examined from pre-conception to six months post-partum. We aimed primarily to determine if women deficient in Vitamin D (<50nmol/L) were more likely to have disordered sleep compared with those Vitamin D sufficient. A secondary aim was to examine if women deficient in Vitamin D were more likely to be depressed or anxious compared with those with adequate Vitamin D levels.

**Methods:** We examined sleep data from women with at least one measurement of Vitamin D in the pregnancy and post-delivery periods (*n=*515). Pittsburgh Sleep Quality Index (PSQI) scores were compared between those with sufficient or deficient Vitamin D levels: by convention a PSQI score of >5 is considered to indicate disordered sleep. Depression was assessed using the Edinburgh Postnatal Depression Score (EPDS), with a score >13 indicating depression. The State-Trait Anxiety short form scale was used to measure anxiety, with a cut off >45 indicating state anxiety. One-way ANOVA in Stata version 18.0 was used throughout.

**Results:** As reported previously, the intervention substantially reduced the proportion of women who were Vitamin D deficient during pregnancy but did not change EPDS scores; PSQI scores were also not changed by the intervention. In the combined control and intervention group total PSQI scores increased from pre-conception until six weeks post-delivery. Total hours of sleep declined from pregnancy weeks 19–20 to six weeks post-delivery. At recruitment preconception, PSQI scores were higher in those deficient in Vitamin D, compared with those with sufficient levels (p=0.015); at later time-points PSQI scores were higher in Vitamin D deficient women but not significantly so. Depression as assessed by EPDS >13 was associated with Vitamin D deficiency only at preconception recruitment (p=0.019) and at 7 weeks’ gestation (p=0.004), but not later in pregnancy or post-delivery. There was no association found between anxiety and Vitamin D status.

**Conclusion:** At pre-conception, sleep was worse in women with low Vitamin D levels. At preconception and early in pregnancy, low Vitamin D levels were associated with depression. Intervention with a Vitamin D containing supplement did not improve sleep in pregnant women.

